# Diagnosis of sudden cardiac arrest using principal component analysis in automated external defibrillators

**DOI:** 10.1038/s41598-023-36011-9

**Published:** 2023-05-30

**Authors:** Van-Su Pham, Anh Nguyen, Hoai Bac Dang, Hai-Chau Le, Minh Tuan Nguyen

**Affiliations:** 1 Data and intelligent systems laboratory, Posts and Telecommunications Institute of Technology, Hanoi, Vietnam; 2 Posts and Telecommunications Institute of Technology, Hanoi, Vietnam

**Keywords:** Biological techniques, Biotechnology, Neuroscience, Biomarkers, Cardiology, Health care

## Abstract

Sudden cardiac arrest (SCA) consisting of ventricular fibrillation and ventricular tachycardia considered as shockable rhythms is a life-threatening heart disease, which is treated efficiently by the automated external defibrillator (AED). This work proposes a novel design of the SAA, which includes a k-nearest neighbors model and a subset of 8 features extracted from the ECG segments, for the SCA diagnosis on the electrocardiogram (ECG) signal. These features are addressed as the most productive subset among 31 input features based on the evaluation of the feature correlation. The recursive feature elimination algorithm combined with the Boosting model and wise-patient fivefold cross-validation method is adopted for the calculation of the average feature importance, which shows the degree of feature correlation, to construct various input feature subsets. Moreover, component feature combinations known as the representatives of the input feature subsets with an enormous level of correlation and independence are transformed from the input subsets by the principal component analysis method. The wise-patient fivefold cross-validation procedure is used for the evaluation of these component feature combinations on the validation set. The proposed SAA is certainly efficient for SCA detection with a small number of the extracted feature and relatively high diagnosis performance such as accuracy of 99.52%, sensitivity of 97.69%, and specificity of 99.91%.

## Introduction

Shockable rhythms including ventricular fibrillation and ventricular tachycardia, known as the abnormal waveforms of the electrocardiogram (ECG) signals, are the main cause of Sudden cardiac arrest (SCA), which results in the death if the life-support services are not provided immediately for the patients. Until now, automated external defibrillator (AED) associated with a shock advice algorithm (SAA) is the most productive device for rapid diagnosis of the SCA and countershock delivery to reset the electrical system of the heart^1^.

Recently, research on SCA diagnosis using intelligent technologies has focused largely on the improvement of the SAA performance. Indeed, incorrect non-shockable or shockable rhythm detection by the SAA leads to no curable solution for the patients who are under the SCA or the defibrillation which causes the artificial SCA. Moreover, machine learning (ML)^1–10^ and deep learning (DL)^11–16^ in combination with advanced signal processing techniques have been widely proposed for the SAA design in terms of AED performance improvement. Generally, the diagnosis performance of the SAA is better than that of the conventional methods, which use different thresholds for SCA classification^10^, and meet the American Heart Association recommendation^17^. The DL-based SAA designs show various advantages in comparison with the ML-based SAAs such as no feature extraction, feature score-based feature ranking, feature selection, and better representative features, which reduces the complexity of the process of SAA designs^12^. However, the identification of the optimal DL models is a time-consuming procedure due to the optimization of different hyper-parameters and structures^12–14,16^. Furthermore, the utility of the DL models requires massive data, which makes it difficult for data collection. Nevertheless, the ML techniques have proven their effectiveness for the SAA designs in terms of model construction with the small amount of data, better adaptation to binary classification, and short time consumed for the model optimization^2,3,5,6,10^.

To improve the classification performance of the SAA designs using the ML or DL methods, different algorithms have been used for signal processing such as ensemble empirical mode decomposition (EEMD)^4^, discrete wavelet transform^5^, variational mode decomposition (VMD)^6,8,10,12^, Taylor Fourier transform^7^, stationary wavelet transforms^9^, fixed frequency range empirical wavelet transform (FFREWT)^13^. The rationale behind the use of these techniques is the increase in the ECG signal quality, which results in better-extracted features. Indeed, subsignals, which are generated from the original ECG signals by the above techniques, contain properly the non-shockable and shockable components of the original ECG segments. Therefore, representation for recognizing the non-shockable and shockable components is better for the input features extracted from the subsignals in comparison with that extracted from the original ECG segments. Moreover, the feature selection methods, which are genetic algorithm^1^, differential evolution algorithm^2^, Gaussian genetic algorithm^3^, correlation attribute evaluation^4^ are also employed to address the most relevant features from the input features. In other words, the important responsibility of the feature selection algorithms is the identification of the optimal features, which are uncorrelated certainly. The diagnosis performance of the ML- and DL-based SAA designs is negligible using the advanced signal processing techniques to improve the quality of the extracted features as shown in Refs.^5–10,12,13^, respectively. We name the application of the advanced signal processing techniques for the achievement of better feature quality as the ECG signal transformation with respect to the SAA designs in this paper.

Principal component analysis (PCA) is a well-known method for the extraction of robust features from the non-stationary ECG signals, which are associated with the rapid property changes of the waveforms. Indeed, the correlation between input features plays an essential role in the selection of the most informative features. It is clear that the utility of the PCA offers a robust approach for the feature estimation based on their correlation degrees^18^. Particularly, the input features are transformed into another space by the PCA, in which the principal components corresponding to the correlation degrees are ordered. Consequently, the featured representative includes a feature subset selected from the input features, which are highly uncorrelated degrees.

Motivated by that the principal components transformed from the input features by the PCA can be served as the alternative input features, which are possibly contributive to the improvement of the proposed SAA design. Moreover, the feature transformation, in which the principal components are used as the alternatives for the input of the ML and DL algorithms, has not been considered properly in previous works related to the shockable/non-shockable rhythm classification. In this paper, a novel SAA design is proposed for the SCA detection from the ECG signals using the K-nearest neighbor (KNN) model and a component feature combination (CFC). First, the feature subset is selected carefully by the recursive feature elimination (RFE) method from the input features using the feature importance computed by the Boosting (BS) model and wise-patient fivefold cross-validation (CV) procedure. Second, the feature transformation is implemented by the PCA for the construction of the CFCs, which is then used for the model optimization on the training set. Last, the ML model using the CFCs is estimated its diagnosis performance by the wise-patient fivefold CV method on the validation set. The main contributions of this work are as follows:

- Evaluation of the feature correlation by the use of feature importance in the original feature space and generation of high feature uncorrelation by the PCA transformation for the construction of the alternative CFC in the principal component space.
- The utility of average important values, which are computed repeatedly by the RFE algorithm including a BS model in combination with the CV method leading to a reliable estimation for the feature selection.
- Proposal of a simple and effective SAA for the AED, which exposes relatively high performance with respect to the SCA detection.

## Data and preprocessing

**Figure 1:**
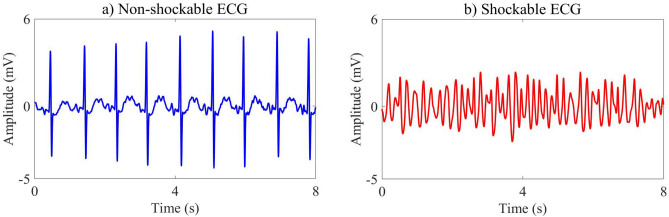
Non-shockable and shockable 8s-ECG segments.

The public databases, which are the Creighton University Ventricular Tachyarrhythmia Database (CUDB) and the MIT-BIH Malignant Ventricular Arrhythmia Database (VFDB), are considered for method development and validation in this study^19^. The CUDB and VFDB include 35 single- and 22 double-channel records in which record lengths are 8 and 35 min, respectively. The ECG signal annotations of VF, VT, and ventricular flutter are annotated as shockable signals, whose bandwidth is ranged from 0 to 10 Hz^10^, while non-shockable signals contain other types of ECG signals such as normal sinus, paced, nodal rhythms, atrial fibrillation, and ventricular ectopic beats. For the achievement of a better learning process, only the first channel of the VFDB is employed for this work. A sampling frequency of 250 Hz is applied for a total of 57 records of the ECG databases, which are then divided into non-overlapping 8s segments. The sampling frequency of the databases is 250 Hz. A total of 57 records are then separated into non-overlapping 8-s segments of the ECG signals. Due to no contribution to SCA diagnosis, noise, asystole, transition rhythms, slow VT rate under 150 beats per minute of intermediate rhythms, and VF rhythms with peak-to-peak amplitude under 200 $\upmu$V are eliminated from the ECG records^12^. Removal of the asystole, which is considered as NSH rythm, based on the zero amplitude of the ECG signals provides appropriate requirements for the SAA. As a result, 1135 shockable and 5185 non-shockable segments are further used for this work. Moreover, wise-patient separation, also known as inter-patient paradigm, is implemented to divide the records coming from different patients into training and validation sets. Indeed, 40 and 17 records collected from the individual patients, which account for 70% and 30% of the entire databases corresponding to 4303 and 2017 segments, are assigned for training and validation sets, respectively. The records, shockable, and non-shockable ECG segments of CUDB and VFDB databases divided into training and validation sets are given in Table 1. Figure 1 shows the shockable and non-shockable ECG segments, which are preprocessed as follows:

(i) Generation of the smooth ECG signal by five-order moving average filtering.
(ii) Removal of drift suppression and baseline wander by high-pass filtering with 1 Hz cutoff frequency.
(iii) Elimination of high-frequency interference by second-order 30 Hz low-pass Butter-worth filtering. It is noteworthy that the existing AED supports a bandwidth of 1–30 Hz for monitor-type ECG^6^.

**Table 1:** Number of records, shockable and non-shockable ECG segments in training and validation sets.

Database	Training set				Validation set				Total		
	Record	Shock	Non-shock	Total	Record	Shock	Non-shock	Total	Record	Shock	Non-shock
CUDB	25	292	1056	1348	10	54	330	384	35	346	1386
VFDB	15	642	2313	2955	7	147	1486	1633	22	789	3799
Total	40	934	3369	4303	17	201	1816	2017	57	1135	5185

## Method development

**Figure 2:**
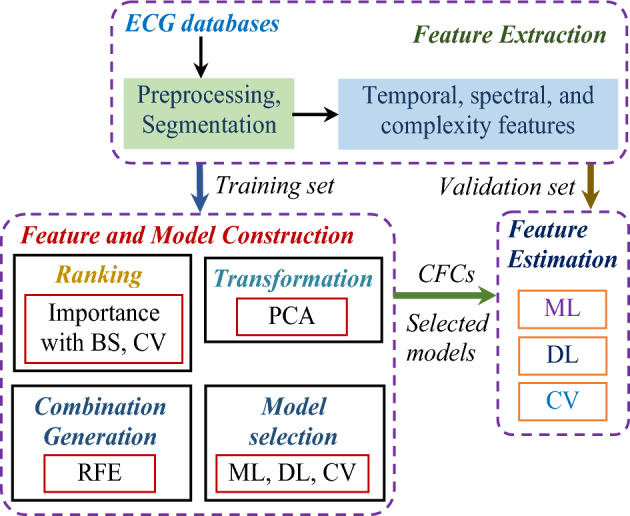
Flow of method development.

The proposed method is shown in Fig. 2 including three main stages. The feature extraction is implemented firstly for the collection of the input features in time and frquency domains. Here, the original ECG signals are divided into segments, which are then preprocessed by various techniques. Secondly, the recursive feature elimination algorithm is performed for the construction of different feature subsets using the importance computed by the BS model. Then, above feature subset is transformed into the CFCs in the principal domain, where the model selection and the SCA classification are completed. Lastly, the wise-patient fivefold CV procedure is employed for the validation of the selected ML and DL models using a number of the CFCs on the validation set. A feature subset corresponding to a component feature combination, which is adopted as the input of a classifier producing the highest validated diagnosis performance in terms of accuracy, is chosen as the proposed design of the SAA. All the principal components of the CFCs corresponding to IFSs are used as the input of ML and DL models. In addition, relatively high diagnosis accuracy is essential to ensure the reliability of the proposed algorithm for the application in the clinic environment. Therefore, we propose a criteria of accuracy, which is larger than 99%, for the selection of the proposed SAA.

We use 6 ML and 2 DL algorithms for this works, which are KNN, Support vector machine (SVM), Boosting (BS), Bagging (BG), Random forest (RF), Logistic regression (LR), Convolutional neural network (CNN), Long-short term memory (LSTM)^20^. It is noteworthy that the tSNE is unconsidered as the classification model in this work due to unstable and unlabelled outcomes, which are unfitted for the simulation using CV method.

### Feature extraction

The input features are extracted from the preprocessed ECG segments by application of various conventional techniques, which are investigated from a large number of the existing publications. These features, also known as the threshold algorithms, are the most common used for the shockable/non-shockable rhythm classification. Indeed, each feature is an algorithm for the calculation of a threshold using to distinguish shockable from non-shockable ECG segments. However, the thresholds are unnecessary when the outputs of the corresponded algorithms are used as the input features of the ML or DL methods^10^. Moreover, the feature contribution is only witnessed by their correlation within a completed feature combination for practical SCA diagnosis. There is a total of 31 features extracted from the 8-s ECG segments including temporal, spectral, and complexity features^10^ as follows:

- Temporal features: mean absolute value (MAva), bCP, threshold crossing sample count (TCSC), threshold crossing interval (TCin), modified exponential algorithm (MEal), standard exponential algorithm (SEal), Count1, Count2, and Count3.
- Spectral features: center frequency (CFre), spectral analysis (A1 and A2), power spectral analysis (PSan), center power (CPow), Y$\_$Li, bWT, bW, VF-filter leakage measure (VFLM).
- Complexity features: Hilbert transform (HTra), covariance calculation (CCal), phase space reconstruction (PSre), area calculation (ACal), frequency calculation (FCal), Kurtosis (Kurt), complexity measure (CMea), dispersion entropy (DEnt), sample entropy (SEnt), energy (Ener), Renyi entropy (REnt), fuzzy entropy (FEnt), and wavelet entropy (WEnt).

The mean values of features calculated for 50 non-shockable and 50 shockable ECG segments are totally different as shown in Table 2. A total of 31 features is possibly categorized into two groups using the differences between average feature values computed for shockable and non-shockable ECG segments. These feature groups consist of 17 and 14 features, which produce large and small differences between the average values of shockable and non-shockable ECG segments. Intuitively, difference between the values is proportional to feature capability of the recognition with respect to the shockable/non-shockable ECG segments.

**Table 2:** Mean values of features for non-shockable and shockable ECG segments.

Feature	ECG segment		Feature	ECG segment		Feature	ECG segment	
	Non-shockable	Shockable		Non-shockable	Shockable		Non-shockable	Shockable
MAva	0.00	0.65	A1	0.01	1.60	PSre	0.00	0.18
bCP	0.00	0.15	A2	0.00	0.45	ACal	3.02	1462.45
TCSC	0.01	55.87	PSan	0.32	3.48	FCal	0.01	3.14
TCin	2.69	298.82	CPow	0.00	0.04	Kurt	0.05	− 0.06
MEal	0.15	199.88	Y_Li	0.00	47.63	CMea	0.00	0.17
SEal	0.26	192.00	bWT	0.00	0.53	DEnt	0.00	1.60
Count1	0.04	42.96	bW	0.02	1.68	SEnt	0.00	0.35
Count2	0.16	102.33	VFLM	0.00	0.48	Ener	0.47	224.07
Count3	0.01	30.97	HTra	0.00	0.19	REnt	0.01	4.67
CFre	0.32	3.48	CCal	0.00	0.20	FEnt	0.00	0.05
						WEnt	0.00	0.96

### Feature and model construction

#### Feature construction

In this stage, we use the RFE method for the construction of various feature subsets, which are then transformed into component space by the PCA. Firstly, the input features are ranked by their importance values, which are computed by the BS model in combination with the wise-patient fivefold CV procedure. Here, the features, which have largely average importance values, are considered as more important than the others. Then, the feature subsets are constructed by the removal of the features with the lowest average importance values. It is noteworthy that the BS model combined with the wise-patient fivefold CV procedure is implemented repeatedly to calculate the feature importance for every feature subsets until no feature for subtraction. Algorithm 1 represents the RFE method for the construction of different feature subsets.


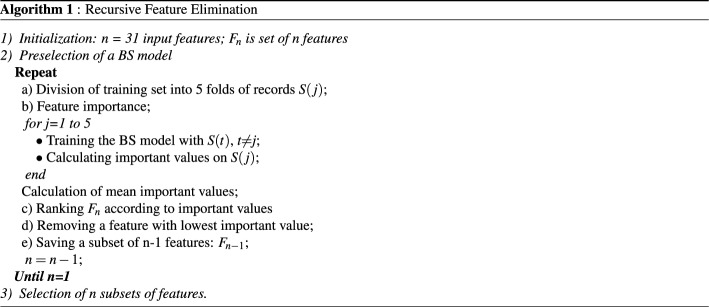


##### Feature importance

Given that *n* features, denoted as $F_{n}$, are include in the input data *X*. A relevant measurement for each feature is computed as follows^21^:

1 $$\begin{aligned} R_{n}^2(Tr) = \sum \limits _{i=1}^{T-1} \hat{s}_{i}^2 I(v(i)=n), \end{aligned}$$

where *I* is the indicator of set *v*(*i*). *Tr* is a single tree, which consists of $T-1$ internal nodes. A region associated with a node *v*(*i*) is partitioned by an input variable $F_n(v(i))$ into two subregions in which the separated constants are fit to the response values. The maximal estimated improvement $\hat{s}_i^2$ in squared error risk is given by the above variable, which results in a constant fitting over the entire region. The importance of variable $F_{n}$ is the sum of the improvement over all internal nodes.

For calculation of $\hat{s}_i^2$, we note that a system contains a random output, known as response, variable *y* and the input or observations $x = \{x_1, x_2,..., x_K\}$. $p_t(x) = P(y_t|x)$ is the conditional probability of *x* given *y* with $y_{t} \in \{0, 1\}$, $t \in \{t_{1}, t_{2}\}$ are the binary classes, and $k \in \{1 : K\}$. *L* and *P* are the numbers of trees and iterations, respectively. Each of tree has $T-1$ internal nodes corresponding to regions $\{Z_{itp}\}_{l=1}^{L}$. *L*-trees are made at each iteration *p* to predict the corresponding residuals for each class *t* on the probability scale. The maximal estimated improvement $\hat{s}_i^2$ is used to estimate the splits of region $\{Z_{itp}\}$ into two subregions corresponding to class $t_{1}$ and $t_{2}$ as follows:

2 $$\begin{aligned} \hat{s}_i^{2}(t_{1},t_{2})=\frac{w_{t_{1}}w_{t_{2}}}{w_{t_{1}}+w_{t_{2}}} (\overline{y}_{t_{1}}-\overline{y}_{t_{2}})^{2}, \end{aligned}$$

where pseudoresponses $\overline{y}_{t_{1}}$ and $\overline{y}_{t_{2}}$ are the means of left and right daughter responses. $w_{t_{1}}$ and $w_{t_{2}}$ are the corresponding sums of weights. These are computed with respect to observation $x_{k}$ as follows:

3 $$\begin{aligned} \overline{y}_{kt}=\frac{L-1}{L}\frac{y_{kt}-p_{t}(x_{k})}{p_{t}(x_{k})(1-p_{t}(x_{k}))}, \end{aligned}$$

4 $$\begin{aligned} w_{t}(x_{k})=p_{t}(x_{k})(1-p_{t}(x_{k})). \end{aligned}$$

The importance measure is simply averaged over the tree based on the generalization of the additive tree expansions as follows:

5 $$\begin{aligned} R_{n}^2 =\frac{1}{L} \sum \limits _{l=1}^{L} R_{n}^2(Tr_{l}). \end{aligned}$$

For binary classification, two separate models $M_{1}(x)$ and $M_{2}(x)$ are generated in which each model contains a sum of trees as follows:

6 $$\begin{aligned} M_{1}(x) =\sum \limits _{l=1}^{L} Tr_{1l}(x), \end{aligned}$$

7 $$\begin{aligned} M_{2}(x) =\sum \limits _{l=1}^{L} Tr_{2l}(x). \end{aligned}$$

Then, the Eq. (5) is generalized for binary classes as follows:

8 $$\begin{aligned} R_{n1}^2 =\frac{1}{L} \sum \limits _{l=1}^{L} R_{n}^2(Tr_{1l}), \end{aligned}$$

9 $$\begin{aligned} R_{n2}^2 =\frac{1}{L} \sum \limits _{l=1}^{L} R_{n}^2(Tr_{2l}). \end{aligned}$$

The final relevance of $F_{n}$ is computed as the mean of two classes as follows:

10 $$\begin{aligned} R_{n}^2 =\frac{1}{2}(R_{n1}^2+R_{n2}^2). \end{aligned}$$

Here, the utility of binary classification results in relevances of $F_{n}$ related to the observations of the individual class separated from another. Hence, $R_{n1}$ and $R_{n2}$ are the relevances of $F_{n}$ corresponding to two classes.

Obviously, the importance measures are relative and obtained by the respective square roots as shown in Eqs. (1) and (5). Therefore, the largest importance value is customary assigned as 100 to scale the other values accordingly.

##### Principal component analysis

Different feature subsets, which are generated by the RFE using the BS model and the wise-patient fivefold CV, are transformed into component space by the PCA in which each feature subset corresponds to a CFC. Obviously, high correlation between features in the original feature subset is the main reason for the utility of PCA transformation to obtain the component subset in which individuals are uncorrelated significantly.

More precisely, the training set with *n* features, known as observed variables $F_{n}$, is transformed into a different space including *n* principal components $C_{n}$, which are independent and uncorrelated variables. First, the features are standardized to make them independent on measurement scale. Then, a feature is given as follows:

11 $$\begin{aligned} F_{m} = a_{m1}C_{1}+a_{m2}C_{2}+ \cdots +a_{ml}C_{l}+ \cdots +a_{mn}C_{n}. \end{aligned}$$

Also, the principal components, which are used as the CFC for further model selection and feature estimation, are computed as a linear combination of the original features as follows:

12 $$\begin{aligned} C_{l} = b_{1l}F_{1}+b_{2l}F_{2}+ \cdots +b_{ml}F_{m}+ \cdots +b_{nl}F_{n}. \end{aligned}$$

The component $C_{l}$ are uncorrelated with each other and ordered by the sample variance $\lambda$, that is, the largest sample variance represents for $C_{1}$, the second largest sample variance is for $C_{2}$, and so on. The sample variances corresponding to different components are known as eigenvalues, which show the share proportion in the total variance. In details, a component including a larger share of the total variance, which represented by a larger eigenvalue, in the original features is more important than the others with smaller eigenvalues. The covariance matrix *Cov* of the original feature *F* is as follows:

13 $$\begin{aligned} Cov=\begin{bmatrix} 1 &{} x_{12} &{} ...&{} x_{1j} &{} ...&{} x_{1n}\\ x_{21} &{} 1 &{} ... &{} x_{2j} &{} ...&{} x_{2n}\\ . &{} . &{} ... &{} . &{} ... &{}.\\ x_{i1} &{} x_{i2} &{} ... &{} x_{ij} &{} ... &{} x_{in}\\ . &{} . &{} ... &{} . &{} ... &{}.\\ x_{n1} &{} x_{n2} &{} ... &{} x_{nj} &{} ... &{} 1 \end{bmatrix}, \end{aligned}$$

where $x_{ij}$ is the correlation of $F_{i}$ and $F_{j}$. The coefficients $b_{ml}$ with $m = [1:n]$ for component $C_{l}$ contain the eigenvector corresponding to the $l{\text {th}}$ largest eigenvalue $\lambda _{l}$. Due to standardization, the total variance of all features equals to the number of features as follows:

14 $$\begin{aligned} n = \lambda _{1} + \lambda _{2} + \cdots + \lambda _{n}. \end{aligned}$$

Hence, $\lambda _{l}/n$ is the proportion of the total variance for the $l{\text {th}}$ component. It is noteworthy that the similar number of features and components remains the entire information during transformation process.

#### Model selection

All the CFCs are fed into different ML and DL models to search for the optimal learning and structure parameters on the training set. Obviously, hyper-parameter tuning is necessary for the identification of the optimal models, which contributes significantly to the avoidance of the overfitting problem. Furthermore, the selected models corresponding to the CFCs are estimated for their classification performance on the validation set. In this work, we use the grid search in combination with the wise-patient fivefold CV method to address the optimal parameter values for the models using entire CFCs.

### Feature estimation

The optimal models, which are the output of the model selection phase, are estimated the detection performance on the validation set using the CFCs. In addition, the wise-patient fivefold CV procedure is also implemented to obtain reliable simulation results. There are two steps of the above procedure, which are data separation and model validation. For the former, the validation set is separated into 5 subsets of the records in which a subset is the testing data while others are used for training the models. Training and testing of the models are implemented repeatedly 5 times to make every subset become the testing data in the latter. The wise-patient CV procedure is run 30 times to compute the mean and standard deviation of the classification performance. A final feature subset corresponding to a CFC used as the input of the model, which produces the highest detection accuracy among others, is selected. The SAA design including such a final feature subset and the above model is proposed for the practical AED.

## Results

### Performance measure

A number of measures namely accuracy (Ac), sensitivity (Se), specificity (Sp), and balanced error rate (BER) are used for performance estimation of the ML models using the CFCs. The Ac measures the number of ECG segments identified correctly. The shockable and non-shockable ECG segments addressed correctly are represented by the Se and Sp. BER is computed as 1 − 0.5(Se + Sp).

**Figure 3:**
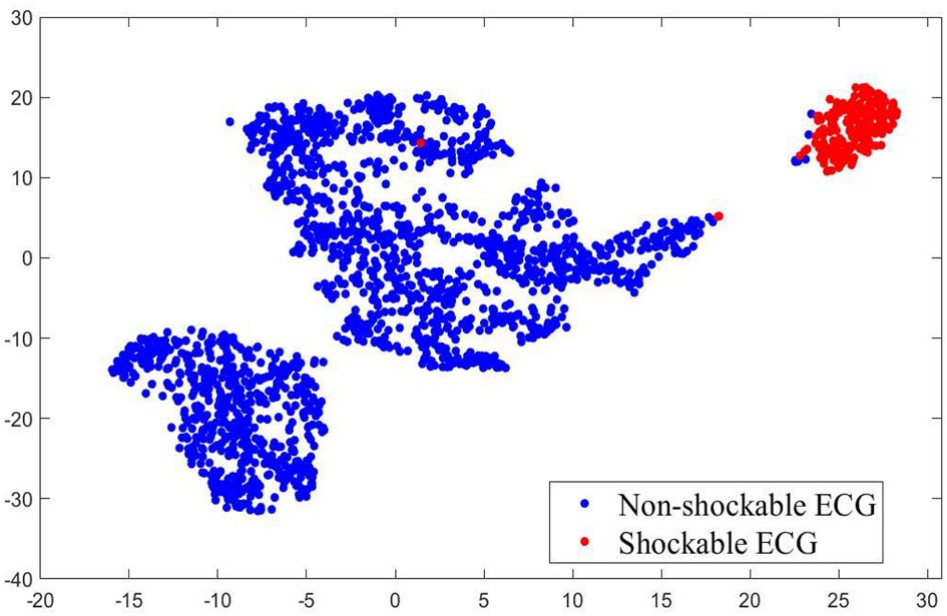
tSNE visualization of validation set with a CFC transformed from an IFS of 8 features.

### Feature and model construction

#### Feature construction

A total of 31 input features, which are extracted from the preprocessed ECG segments, are adopted to construct 31 feature subsets in which the number of features is from 1 to 31 using the RFE method. Moreover, the BS model and wise-patient fivefold CV procedure, which are included in the RFE algorithm, are applied 30 times to produce 30 tables of the important values of the individual feature subsets. Then, the features with the lowest important values in the above tables are removed. All the feature subsets are transformed into component space by the PCA for the generation of 31 CFCs. Figure 3 shows the tSNE visualization of the validation set with a CFC corresponding to an input feature subset (IFS) of 8 features. The individual input features are ranked by the RFE algorithm as shown in Table 3. The selected times of the individual features by the RFE method are represented by the Imp number.

**Table 3:** Individual feature ranked by RFE method.

Feature	bCP	VFLM	Count2	SEnt	Ener	FCal	bW	Y$\_$Li		MEal	bWT
Imp	30	29	28	27	26	25	24	23		22	21
Feature	Count1	REnt	Kurt	FEnt	TCSC	A2	CMea	Count3	PSre	TCin	DEnt
Imp	20	19	18	17	16	15	14	13	12	11	10
Feature	CPow	WEnt	CCal	HTra	A1	PSan	MAva	SEal	ACal	CFre	
Imp	9	8	7	6	5	4	3	2	1	0	

#### Model selection

A total of 8 ML, DL algorithms, and 31 feature subsets corresponding to 31 CFCs are considered in this work. Hence, there are a total of 248 models, which are optimal for the individual feature subsets using the grid search method in combination with the wise-patient fivefold CV procedure.

### Feature estimation

There are 248 models in the total using the CFCs, which are then estimated their diagnosis performance on the validation set by the wise-patient fivefold CV procedure. Table 4 shows 8 models, which produce the highest detection accuracy and the number of features in the corresponding IFSs. Particularly, each model in Table 4 is chosen by the classification accuracy comparison of 31 models using 31 CFCs, which are transformed from 31 IFSs by the PCA.

**Table 4:** The highest detection performance of individual models using the input of the CFCs, which are transformed from the IFSs including different number of original features.

Model	# feature	Ac (%)	Se (%)	Sp (%)	BER (%)
SVM	4	99.45 ± 0.24	97.93 ± 0.86	99.57 ± 0.18	1.24 ± 0.42
LR	4	99.17 ± 0.23	96.97 ± 0.49	99.37 ± 0.13	1.83 ± 0.29
*KNN*	*8*	*99.52* ± *0.01*	*97.69* ± *1.23*	*99.71* ± *0.03*	*1.30* ± *0.63*
RF	26	99.22 ± 0.09	96.26 ± 1.82	99.37 ± 0.15	2.16 ± 0.90
BG	26	99.08 ± 0.14	95.98 ± 1.18	99.20 ± 0.19	2.41 ± 0.59
BS	26	99.49 ± 0.16	95.17 ± 1.86	99.74 ± 0.16	2.54 ± 0.99
CNN	6	99.40 ± 0.10	95.39 ± 2.41	99.73 ± 0.09	2.44 ± 1.25
LSTM	5	99.18 ± 0.78	98.06 ± 0.43	99.34 ± 0.10	1.34 ± 0.26

Significant values are in italics.

### Proposed SAA

The SAA design, which is proposed for the AED in this work, contains a KNN model (K = 15) and a subset of 8 features extracted from the ECG signals with a length of 8 s. Here, the KNN model combined with CV procedure is implemented repeatedly with different K values ranging from 5 to 100. The value of K for which the KNN model produces the highest detection accuracy is selected as the optimal parameter. The proposed SAA is installed on the AED to detect the SCA as follows:

(i) Collection of an ECG segment with a length of 8s using a slipped window and the electrode pads of the AED.
(ii) Implementation of the preprocessing techniques to obtain clean the ECG signal.
(iii) Extraction of an IFS including 8 features from the clean ECG signal.
(iv) Transformation of the above IFS into principal component space by the PCA to obtain the CFC.
(v) Assigning the labels to such ECG segment as 0 for non-shockable ECG signal or 1 for shockable ECG segment by the trained KNN model using a CFC transformed from an ICF of 8 features.

The average duration of the segmentation, signal preprocessing, feature extraction, feature transformation, and classification consumed by the proposed SAA is calculated for 50 consecutive ECG segments as $3.97 \pm 2.28$. Certainly, this total time is shorter than the ECG segment length of 8s, which ensures no interruption of the SCA diagnosis between the consecutive ECG segments. According to AHA recommendations, a minimal number of 3 ECG leads, also known as 3-wire lead set, is employed for the ECG acquisition. Two leads are right arm (RA), left arm (LA) electrodes placed under right and left clavicles, mid-clavicular line within the rib cage frame. The last is left leg (LL) electrode, which is placed on the lower left abdomen within the rib cage frame.

## Discussion

Nowadays, the AED, which is convenient, low-cost, and reputable, is the most productive decision-support system for SCA treatment. Indeed, the shockable rhythms are diagnosed reliably on the ECG signals by the SAA, which is integrated into the AED. Then, rapid defibrillation is delivered to start the electric system of the heart over, which improves the chance of survival.

The feature correlation plays a vital role in the estimation of the feature quality for the classification of the shockable/non-shockable rhythms on the ECG signals. Indeed, the overlapping degree of the information, which is carried by the features, is measured by the feature correlation or feature importance. Hence, the individual features should contain the separated information, which can be used to discriminate correctly the shockable from non-shockable ECG segments.

Evaluation and generation of the feature correlation are considered for the feature selection, which consolidates the representatives of the selected features in the final CFC. Basically, a feature is removed by the RFE method using the important value computed by the BS model in combination with the wise-patient fivefold CV method from the input features to construct a new IFS. Here, the mean values of the feature importance are calculated repeatedly for every IFS to address a feature with the lowest mean value of the importance for the elimination. This procedure aims at the correlation evaluation between features in an IFS when the feature number is changed. Consequently, the persistent features, which are preserved after the repetitions of the RFE algorithm, are highly uncorrelation with others. Another significant characteristic is that the generation of the feature correlation is implemented by the PCA transformation. Indeed, each CFC includes highly uncorrelated features, also known as the principal components, which are proven by Eq. (2). According to such formulation, each principal component is constructed from different fractions of all input features in a specific IFS. Obviously, the information related to shockable/non-shockable rhythms is accumulated to form a new feature namely the principal component. The highly validated performance of the ML and DL algorithms in Table 4 shows the impressive effectiveness of the proposed feature construction in this work. A CFC corresponding to 8 input features, in which individual principal components contain the separated information for the representation of the shockable/non-shockable rhythms on the ECG segments, is significantly efficient as the input of the KNN model for the SCA detection. Table 5 shows the performance comparisons of the proposed SAA with existing algorithms related to the SH/NSH rhythm classification.

Clearly, the selected feature number of the proposed SAA is relatively small in comparison with that of other publications, while the highly validated performance is maintained. Compared to^4,8,10,13^ using the signal transformation method, the feature transformation used for the proposed SAA is simple and powerful. Definitely, the difference between the validated performance of our SAA and that of Ref.^10^, which is the highest as given in Table 4, is negligible, while the proposed SAA design uses only a small number of 8 input features compared with 36 features determined in Ref.^10^.

**Table 5:** Performance comparisons of the proposed SAA with existing works.

Ref., year	Approach	# Feature	Ac (%)	Se (%)	Sp (%)
^2^, 2021	AdaBoost	17	98.20	98.25	98.18
^4^, 2021	C4.5, EEMD	24	98.69	97.74	99.00
^8^, 2020	C4.5, VMD	24	99.18	97.07	99.15
^10^, 2022	SVM, VMD	36	99.56	98.22	99.84
^13^, 2020	CNN, FFREWT		99.03	99.95	95.95
^16^, 2020	CNN			99.5	99.4
Proposed SAA	KNN, PCA	8	99.52	97.69	99.71

Furthermore, the clinic physiology meanings of the selected input features provide deep knowledge of the shockable/non-shockable rhythms related to SCA diagnosis using the ECG signals. The Count2 feature is defined as the sample number, which represents the clear QRS peaks of the ECG signals. The non-shockable and shockable ECG segments have clear QRS peaks and no peak, which results in small and large numbers of samples for Count2, respectively^22^. The differences between auto-correlation and cross-correlation of the ECG waveforms are computed as the Y_Li feature using continuous wavelet transform. Here, small differences are associated with the clear QRS complexes on the non-shockable ECG segments while no complex is available on the shockable ECG signals leading to large difference values^23^. An essential characteristic of the ECG signals is the regularity, which is measured by the SEnt feature. Large differences in the samples are frequently shown on the shockable ECG segments due to no QRS complex, which leads to a great SEnt value. In contrast, non-shockable ECG signals with a huge number of clear QRS complexes indicate a high level of signal self-similarity, which results in a small value of the SEnt^24^. Given that the ECG signal is similar to the quasi-sinusoidal waveform, a version of the ECG segment is generated by shifting such ECG signal by half a period. A combination of the original ECG segment and its version is the VFLK output, which indicates the small or high values of the amplitude for the shockable or non-shockable ECG segments, respectively^25^. The FCal feature is based on the complexity and disorganization of the ECG signals, which are first converted into binary signals. Well-organized ECG segments are frequently stuck to the isoelectric line, which results in the stability of the binary signals with low FCal values. Conversely, rapid changes of the binary signals for the shockable ECG segments show high values of the FCal^26^. Clear QRS complexes of the non-shockable ECG signals result in high values of the signal slope, which are represented by a large bCP. In contrast, no impressed slope on the shockable ECG signal implies a small bCP value^27^. The broadband and narrowband are assumed for the non-shockable and shockable ECG signals, whose frequency difference is measured by the bW feature. Hence, large and small values of the bW are for non-shockable and shockable ECG segments, respectively^27^. The non-shockable ECG signals are close to the isoelectric line most of the time while a large number of disorganized peaks are shown on the shockable ECG segments. Therefore, the Ener feature values are small and larger for the non-shockable and shockable ECG segments, respectively^28^.

The limitations of our study are the time consumption and complexity of the RFE algorithm using wise-patient fivefold CV procedure, which is run repeatedly to compute the importance values for feature elimination. In addition, the input data are likely small, which reduces possibly the effectiveness of the proposed SAA using online ECG signals in the clinic environments. Subsequently, evaluation of the proposed SAA with massive and diverse ECG databases is open for the future works.

## Conclusion

The change of survival is largely dependent on the rapid, correct diagnosis and countershock delivery of the AED for the SCA on the ECG signals. Moreover, the SAA is the most important element of the AED, which plays the role of the decision-support algorithm. Hence, the performance improvement of the SAA design is paid intensive attention from the medical experts due to high classification performance resulting in avoidance of numerously unexpected deaths.

In this work, we proposed an effective and simple SAA for the AED, which is a potential application for the clinic environment, using the ML technique. The proposed SAA was designed with a KNN model and a subset of 8 input features, which were carefully selected by the RFE method in combination with the BS model and the wise-patient fivefold CV procedure. The feature correlation was evaluated repeatedly for the entire input features using the average feature importance computed by the BS model to eliminate the individual features with the lowest important values. Consequently, various IFSs with different feature numbers were constructed and then converted into the CFCs by the PCA, which were independent and uncorrelated variables. Obviously, the uncorrelation of the ICFs was increased by the use of the CFCs as the input of the KNN model, which led to the relatively high performance of the diagnosis for the shockable/non-shockable rhythms. The validated classification performance with Ac of 99.52%, Se of 97.69%, and Sp of 99.91% on the validation set and average diagnosis time of 3.97 s imply the effectiveness and simplicity of our proposed SAA, which are less complexity than others using signal transformation techniques for the feature extraction.

## Data Availability

The datasets generated and/or analysed during the current study are available on the physionet.org. (https://physionet.org/content/cudb/1.0.0/ and https://www.physionet.org/content/vfdb/1.0.0/).
